# Connecting Bone Remodeling and Regeneration: Unraveling Hormones and Signaling Pathways

**DOI:** 10.3390/biology14030274

**Published:** 2025-03-07

**Authors:** Afshan Mehreen, Muhammad Faisal, Bilal Zulfiqar, Deli Hays, Kavishka Dhananjaya, Faiza Yaseen, Yujun Liang

**Affiliations:** 1 Key Laboratory of Evolution and Marine Biodiversity (Ministry of Education), Institute of Evolution and Marine Biodiversity, Ocean University of China, Qingdao 266003, China; afshanmehreen@yahoo.com (A.M.); faisalshah32402@gmail.com (M.F.); delimarmds@gmail.com (D.H.); kavishka.dhananjaya8@gmail.com (K.D.); faiza.yaseen55@gmail.com (F.Y.); 2 College of Marine Life Sciences, Ocean University of China, Qingdao 266003, China; 3 Discovery Biology, Griffith University, Nathan, QLD 4111, Australia; b.zulfiqar@griffith.edu.au

**Keywords:** regeneration, bone regeneration, bone remodeling, axolotl, zebrafish, hormones

## Abstract

Recent advancements in tissue engineering and stem cell science have positioned bone disease treatment as a promising frontier in regenerative medicine. This review explores the hormonal and signaling pathways critical to bone regeneration, with a focus on their clinical relevance. Key endocrine factors, including thyroid hormones (T3 and T4), insulin-like growth factor 1 (IGF-1), bone morphogenetic proteins (BMPs), parathyroid hormone (PTH), calcitonin, and fibroblast growth factor 23 (FGF23), play pivotal roles in bone remodeling by regulating osteoblast activity, bone resorption, and mineralization. These factors primarily act through the *Wnt*/β-catenin, BMP, and FGF signaling pathways, which govern bone repair and regeneration. While animal models, such as axolotls, zebrafish, and *Xenopus laevis*, provide valuable findings about these mechanisms, translating these findings into human applications presents challenges. This review underscores the therapeutic potential of modulating these hormonal networks to enhance bone regeneration while cautioning against possible adverse effects, such as uncontrolled tissue proliferation or metabolic imbalances. By integrating knowledge from regenerative models, this work provides a foundation for optimizing hormone-based therapies for clinical applications in bone repair and disease treatment.

## Simple Summary

Bone diseases and injuries often result in poor healing and long-lasting damage, affecting millions of people worldwide and highlighting the urgency for better treatments. Understanding the mechanisms of how to control the complex process of bone repair and regeneration without unwanted side effects, such as metabolic imbalances or excessive tissue growth, and translating knowledge from animal studies to humans has remained one of the major challenges. This literature review explores the intricate relationship between bone regeneration and bone remodeling, with a particular emphasis on the role of hormones and key molecular factors that may influence the regenerative capacity in model organisms. It examines the signaling pathways and molecular interactions that facilitate a seamless regeneration process. Key endocrine factors such as PTH, calcitonin, and FGF23 play crucial roles in bone remodeling and healing, with potential therapeutic applications. The review underscores both the opportunities and challenges of translating these findings into human regenerative medicine.

## 1. Introduction

Bone regeneration has significantly advanced regenerative medicine, particularly in tackling injuries that affect the human skeleton and degenerative diseases. Model organisms, such as axolotls, zebrafish, and *Xenopus laevis*, play a crucial role in bone regeneration research due to their remarkable ability to regenerate various tissue types, including bones [1]. The axolotl, a remarkable organism, can regenerate an entire limb, spinal cord, or even parts of its brain at any developmental stage without forming scars [2]. This capability makes axolotls an ideal model for studying the molecular and cellular mechanisms underlying bone regeneration. Zebrafish also provide valuable insights into regeneration processes, primarily due to the transparency of their embryos, which allows for direct observation, as well as their genetic manipulability [3]. Additionally, zebrafish serve as a viable model for studying mammalian biology, as some genes and processes, such as *Wnt* and FGF, are conserved across the species [4]. In contrast, *Xenopus laevis* exhibits a decrease in regenerative ability after metamorphosis, making it useful for revealing the hormonal and genetic regulation of regeneration. Its adult form may provide insights comparable to the loss of regenerative capacity observed in mammals [5].

A variety of hormones play crucial roles in regulating numerous body functions, including growth and development. These chemical messengers, produced by various glands, are responsible for maintaining electrolyte balance, metabolism, and reproduction [6]. Understanding the roles of hormones, such as thyroid hormones, IGF-1, and BMPs, in model organisms enhances our comprehension of the fundamental hormonal regulation of bone regeneration [6].

These hormones also regulate key signaling pathways essential for successful regeneration, including the *Wnt*/β-catenin and BMP pathways, which are involved in critical processes, such as cell differentiation, blastema formation, and tissue remodeling [7].

However, challenges arise when translating findings from these models to human medicine. Issues such as off-target effects, including abnormal tissue growth and metabolic disorders, must be carefully managed [8]. Therefore, while these models provide critical insights into potential regenerative therapies, precision and caution are essential when applying these findings to human clinical contexts.

This literature review highlights the importance of model organisms in studying bone regeneration, mainly focusing on hormones and signaling pathways. There is a limited number of review publications on the role of hormones and growth factors in bone regeneration, as indicated by PubMed; this review seeks to address a critical gap in the literature. By offering a comprehensive analysis of their interactions with key signaling pathways that regulate osteoblast and osteoclast activity, we aim to provide timely and valuable insights that advance research in this field.

The primary focus of the review is to investigate the cooperative and antagonistic roles of key hormones and signaling pathways (e.g., *Wnt*/β-catenin, BMP, TGF-β) in orchestrating bone repair and the remodeling processes. Insights gained from regenerative models like axolotls and zebrafish are being examined to develop safe and effective therapies for bone injuries and degenerative diseases in humans. The therapeutic implications and risks of manipulating hormonal pathways (e.g., PTH, IGFs) are assessed in the context of bone regeneration, including potential side effects.

Secondarily, the study aimed to detail the targeted roles of hormones and their regulation of specific genes and proteins during the inflammatory, repair, and remodeling phases of bone healing and remodeling. In addition, it explores how signaling pathways contribute to tissue repair efficiency and proper remodeling. Challenges such as balancing innovation with safety—particularly in mitigating risks associated with hormonal manipulation—are addressed. Evolutionary similarities in regenerative pathways between humans and model organisms are investigated, providing insights that can enlighten the development of precision medicine strategies.

## 2. Model Organisms

Model organisms, like axolotls, zebrafish, and *Xenopus*, are pivotal in regenerative research, particularly for understanding bone regeneration, due to their distinct regenerative capabilities.

- Axolotl: Renowned for its exceptional ability to regenerate various body parts, it serves as a valuable model for investigating the molecular and cellular processes involved in tissue regeneration, particularly in bones [7]. This regenerative capacity persists throughout the axolotl’s life, offering insights into mechanisms that could potentially be reactivated in humans to combat chronic diseases. The axolotl’s capability to regenerate entire structures, such as limbs, makes it an ideal model for studying the life cycle of bone regeneration, from blastema formation to the reactivation of developmental processes [1];
- Zebrafish: Zebrafish are also instrumental in studying bone regeneration, as their ability to regenerate fins involves processes analogous to those in bone healing. Their genetic manipulability and the optical transparency of their embryos allow for direct observation of regenerative events. Zebrafish research has enabled scientists to identify the roles of various genes and molecular mechanisms in bone regeneration, including the *Wnt* and FGF pathways, which are conserved in mammals [8,9,10];
- African Clawed Frog: The African clawed frog is another valuable model for regenerative research, particularly regarding limb regeneration during the tadpole stage. Unlike other model organisms, the regenerative ability of *Xenopus* diminishes as it matures, resembling the regeneration capabilities observed in adult mammals. This regression provides a valuable paradigm for investigating the loss of regenerative capacity, allowing researchers to explore the molecular and cellular mechanisms that regulate this process [5].

While these model systems provide valuable insights into hormonal regulation during regeneration (Table 1), translating this knowledge into human applications looks promising yet challenging.

Table 1 describes the key molecular pathways involved in regeneration across model organisms. It highlights the importance of thyroid hormone levels, which are crucial in transforming to the adult stage during the *Xenopus* life cycle. It also summarizes the IGF-1 role during cell proliferation, BMPs regulating osteogenesis, the vascular endothelial growth factor (VEGF) promoting angiogenesis, and the growth hormone (GH) supporting regeneration. In addition, it also draws attention towards the regenerative and inhibitory effects of retinoic acid (RA). The application of these insights carries the risk of off-target effects, such as excessive cell proliferation, abnormal tissue growth, and metabolic imbalances. This highlights the necessity for precision and care in applying these findings [11]. Additionally, the relationships among various hormones and growth factors remain inadequately understood, necessitating further research to validate the effectiveness of regenerative therapies while minimizing patient risks. The challenge is to balance these highly dynamic restorative processes with their potential hazards, a task that has not yet been fully mastered in therapeutic contexts.

**Table 1:** Hormonal control of key regenerative events in model organisms.

Aspect	Axolotl	Zebrafish	*Xenopus*
*Regenerative Capacity*	Very High—Axolotls’ ability to regenerate complex structures is unmatched, yet the hormonal underpinnings of this capacity remain incompletely understood, especially concerning how these processes might be replicated in mammals [2].	High—Zebrafish are highly efficient at regenerating various tissues, but translating these findings into mammalian models, where similar regenerative abilities are limited, poses a significant challenge [12].	Moderate—The decline in regenerative capacity post-metamorphosis in *Xenopus* suggests a hormonal shift that might be manipulated to extend regenerative potential, though the exact triggers remain elusive [2].
*Thyroid Hormone Role*	Thyroid hormone given exogeneously accelerates limb growth during transition from larva to adult state [13].	The metabolic shifts induced by T3 are substantial for regeneration [14]. Thyroid hormone given exogenously increases the zebrafish pectoral fins differentiation.	Levels of thyroid hormones are highest during metamorphosis. As soon as hind limbs develop, the levels decline in *Xenopus* [2].
*Insulin-like Growth Factor 1*	Promotes proliferation of progenitor cells, ensuring sustained growth [15].	Drives cell proliferation and differentiation [16].	Supports growth during early development; there is a decline in its activity post-metamorphosis [15].
*Bone Morphogenetic Proteins*	Central to bone regeneration, BMPs are critical for osteoblast differentiation [17].	Key regulators of bone formation [17].	Essential for limb development [17].
*Vascular Endothelial Growth Factor*	Less studied in axolotls, VEGF is believed to support vascularization, but the balance between promoting blood vessel growth and avoiding excessive angiogenesis remains a key area for further research [18].	Critical for angiogenesis [18].	Vital for vascular development [18].
*Growth Hormone*	Supports overall growth and regeneration [16].	Enhances overall growth and regenerative capacity [16].	Regulates growth during larval stages [16].
*Retinoic Acid*	Modulates limb regeneration [2].	Involved in fin patterning [17].	Crucial during embryonic development [2].
*Metabolic Regulation*	GH and IGF-1 regulate metabolism to support regeneration [2].	T3 promotes a shift to glycolysis necessary to meet the needs of rapidly dividing cells [14].	Thyroid hormones are central to regulate metabolism during key developmental stages [2].
*Cell Dedifferentiation*	BMPs and T3 drive dedifferentiation [17].	Controlled by BMPs, IGF-1, and RA [17].	Regulated by Activin and BMPs, but decline in dedifferentiation capability after metamorphosis.

Applying these insights carries the risk of unwanted effects, such as excessive cell proliferation, abnormal tissue growth, and metabolic imbalances. This highlights the necessity for precision and care in applying these findings [11]. Additionally, the relationships among various hormones and growth factors remain inadequately understood, necessitating further research to validate the effectiveness of regenerative therapies while minimizing patient risks.

### 2.1. Functions of the Endocrine System in Regeneration

Endocrinology is the branch of medicine and biology that focuses on hormonal study, their functions, and the glands that produce them [19]. The endocrine system plays a central role in coordinating bone regeneration in model species. Model organisms utilize hormonal and genetic interactions to regenerate bones to levels of functionality, maintaining both structural integrity and efficiency.

This study focuses on how the inflammatory response triggered by an injury leads to the formation of a blastema that is driven by the coordinated action of genes and hormones involved in regeneration. Principally, the thyroid stimulating hormone (TSH), IGF 1, BMPs, and some other molecules, such as VEGF and PTH, are involved in metabolism regulation, cell differentiation, and tissue repair [20,21,22,23]. In addition, transcription factors, like Runt-related transcription factor 2 (Runx2) and specific transcription factors, such as *Msx1* and *Pax7*, are vital for maintaining progenitor cells and directing their differentiation into osteoblasts. This underscores the complexity of the hormonal signals and genetic pathways that govern bone regeneration [24,25,26,27]. A deeper understanding of these interactions is essential for advancing regenerative medicine and developing therapies that can effectively replicate these natural processes.

#### 2.1.1. Phases of Bone Regeneration in Axolotl and Zebrafish

##### Initial Injury Response

Immediately after injury, the body must respond to prevent complications and initiate healing. This initial response is crucial in bone regeneration because it lays the foundation for subsequent tissue repair in model organisms. This response typically begins with an inflammatory phase when immune cells are recruited to the injury site to clear debris and initiate necessary signaling pathways for tissue regeneration.

**Figure 1:**
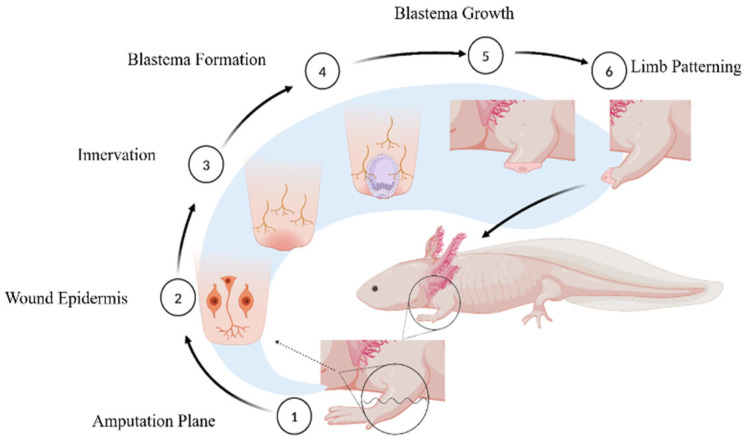
Events followed during Axolotl’s Limb Regeneration. (**1**) Amputation plane where limbs can be amputated to witness the events leading to regeneration. (**2**) Formation of wound epidermis followed by initial immune response for clearing the pathogens and unwanted mass. (**3**) Nerve formation is essential for blastema formation and growth. (**4**) Blastema is the mass of de-differentiated cells necessary for regeneration. (**5**) Blastema cells proliferate and differentiate into cells of choice to develop into new limbs, looking like the original ones. (**6**) Patterning of limbs starts that gives rise to new limbs just like the original ones.

- Hemostasis and Inflammation: The initial response to tissue injury begins with hemostasis, which is critical for limiting hemorrhaging. Following hemostasis, inflammation plays a role in clearing debris and pathogens at the injury site. In zebrafish, this inflammatory phase is characterized by a rapid influx of neutrophils and macrophages, the key components of the innate immune system [28,29,30,31];
  These immune cells act as scavengers, efficiently clearing away damaged cells and debris while releasing a range of cytokines and growth factors that aid tissue repair. Notably, macrophages in zebrafish have the ability to signal and activate progenitor cells, which enhances the healing process [28]. This coordinated immune response is essential for boosting regenerative processes after an injury, ensuring an effective and timely recovery;
  However, in Xenopus, amputation-induced inflammation involves various local physiological changes, such as hypoxia, the generation of reactive oxygen species (ROS), and the release of cytokines that recruit and activate neutrophils and monocytes/macrophages [32]. It has been suggested that the ontogenetic loss of epimorphic regeneration during the transition from the larval stage to adult anuran may be due to changes in the immune system [33]. This may provide a clue to the loss of regenerative capacity in mammals; however, further investigation is needed;
- Activation of Hormonal Pathways: Concurrently with the immune response, or even preceding it, the release of hormones that stimulate tissue regeneration within the endocrine system is initiated. In axolotls, the levels of the hormones triiodothyronine (T3) and thyroxine (T4) remain low throughout their lifespan, indicating that the loss of regenerative ability in amphibians after metamorphosis is driven by the presence of T3 [2].
- Parathyroid hormone related protein (PTHrP) plays a fundamental role in zebrafish skeletal development and regenerative processes through interaction with various receptors, including PTH1R and PTH3R. These receptors are instrumental in stimulating the division and differentiation of osteoblasts, particularly during fin regeneration [34,35]. When PTHrP is secreted in response to injury or developmental signals, it activates osteoblasts essential in bone tissue formation;
  Additionally, transcription factors *Sox9a* and *Sox9b* regulation is critical for cartilage formation, representing a complex regulatory network that fine-tunes the regeneration process [36,37,38,39]. This intricate interplay between PTHrP and the transcription factors highlights the sophisticated biological mechanisms that govern regeneration in zebrafish. Understanding these processes will help to enhance tissue repair and regeneration in other species, including humans, by shedding light on the fundamental aspects of vertebrate regeneration, and it holds promise for developing therapeutic strategies aimed at this;
- Epimorphic regeneration: This is a multi-stage process that begins immediately after amputation, involving complex cellular and molecular interactions to restore lost structures as seen in axolotls (Figure 1). Following amputation, a specialized layer of epidermal cells rapidly covers the exposed area, forming the wound epidermis. This layer plays a crucial role in protecting the injury site, modulating immune responses, and providing essential signals for subsequent regenerative processes. Proper regeneration requires a nerve supply, as nerves release growth factors and signaling molecules that help maintain the microenvironment needed for regeneration. The accumulation of dedifferentiated cells beneath the wound epidermis leads to the formation of the blastema, a mass of highly proliferative progenitor cells. The blastema is the defining feature of epimorphic regeneration and serves as the source for new tissue development, ultimately leading to the complete restoration of the lost structure [40].

In *Xenopus*, thyroid hormone (T3) expression peaks during early development, particularly at the metamorphic stage (refer to Table 1). After the formation of hind limbs and the resorption of the tail, T3 levels decline significantly. This reduction in thyroid hormone levels is hypothesized to contribute to the loss of regenerative capacity observed in *Xenopus* post-metamorphosis [41];

- Blastema Formation
  In both axolotls and zebrafish, the blastema formation is the key for regenerative processes, and the blastema consists of a cluster of undifferentiated cells that act as a central hub for tissue regeneration [42]. This accumulation of cells is essential in the regeneration process of lost or damaged tissues, facilitating the reorganization and differentiation necessary for effective healing. As seen during hind-limb amputation in axolotls, PTH reprograms cells at the wound periphery to a less differentiated state, a crucial step for blastema formation, essential for limb regeneration [43]. This process is mediated via the regulation of key signaling pathways, including *Wnt* and FGF, which govern the primary cellular structures required for successful limb regeneration [44].

On the contrary, *Xenopus* froglet limb amputation leads to the formation of a fibrotic blastema under the wound epidermis, which differentiates partially and later develops into a cone-shaped structure known as a spike [45]. The spike is without a joint or branch; hence, no muscles and ossified bones are developed, which leads to the failure to form patterned limbs despite the initiation of the regeneration process [46].

De-differentiation of Cells: In bone regeneration, osteoblasts are responsible for bone production, and these cells undergo de-differentiation to become multipotent progenitor cells [12,47,48]. This de-differentiation process is essential for forming the blastema, a structure composed of these progenitor cells that will eventually differentiate into new bone and other tissues. Research has shown that the de-differentiation process is tightly regulated by several signaling pathways, including those involving BMPs and thyroid hormones;

- Role of Growth Factors and Hormones: Various growth factors and hormones play crucial roles in blastema formation. For instance, BMP2b and BMP6 are essential for initiating osteoblast de-differentiation and promoting the proliferation of blastema cells in zebrafish. Additionally, BMP signaling is activated by Sonic Hedgehog (Shh) signaling, which not only regulates BMP levels but is also vital for the patterning and growth of the blastema. The Shh pathway mediates this function through the patched I (Ptch1) receptor, which is critical for the formation and differentiation of the blastema. The absence of BMP signaling significantly impairs blastema formation, thereby hindering the regeneration process [17];
- Genetic Regulation: The regulation of blastema formation is another genetic factor that is fundamental for regeneration. In axolotls, Specific transcription factors, like *Msx1* and *Pax7*, show higher expression levels during blastema formation. *Msx1* keeps the cells in an undifferentiated state within the blastema while *Pax7* promotes their proliferation. These Specific transcription factors maintain the blastema as an embryonic pluripotent stem cell reserve that can differentiate into different tissue types, including bone [15].

##### Proliferation and Differentiation: The Journey of Cellular Rebirth

After blastema formation, the cells nestled within embark on a regenerative odyssey, committing to specific pathways of differentiation that are vital for bone formation;

- Cell Cycle Re-entry: The cells in the blastema, eager to bolster the number of progenitor cells, must re-enter the cell cycle. This proliferation is orchestrated by certain growth factors and hormones, with IGF-1 playing a pivotal role. IGF-1 signaling is instrumental in the proliferation of blastema cells, ensuring a sufficient reservoir of stem cells to replace missing bone tissues. Any disruptions in IGF-1 signaling can lead to inadequate cell division, jeopardizing the entire regeneration process [16];
- Metabolic Adaptations: During this proliferative phase, the metabolic demands of the regenerating tissue surpass those of normal tissue [49]. Intriguingly, the blastema cells of zebrafish exhibit metabolic shifts with reduced oxidative phosphorylation and enhanced glycolysis. Controlled by hormones such as IGF-1 and T3, this process is essential to fuel the rapid cell division and maintain the plasticity of progenitor cells. Glycolysis efficiently produces ATP, which powers the high proliferative activity within the blastema [14].
- Differentiation into Bone Tissue: This process not only triggers the proliferation of progenitor cells but also guides their differentiation into osteoblasts, paving the way for bone tissue formation. This transformation is facilitated by BMP signaling, which activates the Runx2, which is crucial for the developmental progression of the progenitor cell lineage and the maturation of these cells into an osteoblast-producing osteoid. In the absence of BMP signaling or a functional Runx2, bone-forming cells cannot differentiate properly, hindering bone remodeling and regeneration [17]. Bone healing reaches its zenith when the newly formed bone tissue undergoes remodeling, ultimately adopting the normal architecture, size, composition, and mechanical properties essential for its function [50,51,52].

## 3. Bone Remodeling: A Journey to Restoration

Bone remodeling is a dynamic process involving the continuous replacement of old bone with new tissue as seen in humans throughout their lives. This process depends on the interaction of various cell phenotypes and is influenced by diverse biochemical and mechanical factors [44]. Bone remodeling starts when signals like mechanical stress or hormones (e.g., PTH) trigger osteocytes. If damage occurs, osteocytes die, leading to the activation of osteoclast formation, which is called the activation phase for bone remodeling. Osteoclasts are recruited and break down old bone by dissolving minerals and digesting the organic matrix. This creates small pits on the bone surface, leading to bone resorption. Followed by resorption, specialized cells clean up leftover bone debris and prepare the surface for new bone formation depositing a thin collagen layer for the reversal of bone resorption. Finally, osteoblasts create new bone by producing collagen and other proteins. These are mineralized to form strong, healthy bone tissue. This cycle ends with the replacement of old damaged bone to maintain healthy, strong bones [53].

**Figure 2:**
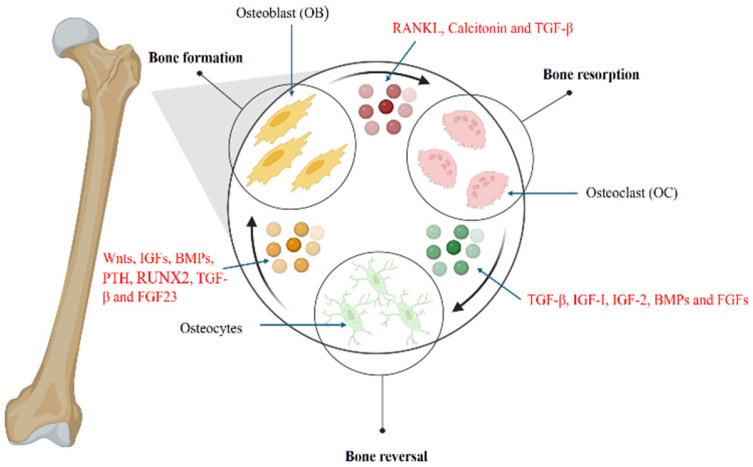
Bone Remodeling: Hormonal regulation of signaling pathways and molecules involved. Bone resorption: Receptor Activator of Nuclear factor Kappa beta Ligand (RANKL) calcitonin and TGF-β are involved in the activation of osteoclasts, promoting bone resorption. Reversal: Resorption is followed by a reversal process where TGF-β, IGF1, 2, BMPs, and FGFs play a central role and activate osteocytes. Bone formation: After activation, osteocytes lead to bone formation involving molecules including *Wnt*, IGF, BMP, PTH, RUNX2, TGF-β, and FGF 23.

As illustrated in Figure 2, this intricate process highlights the multitude of molecules orchestrating the lifelong mechanism of bone remodeling in humans, which parallels the regeneration process observed in model organisms. Each of these molecules plays a critical role in maintaining the structural integrity and functional capacity of the newly formed bone tissue.

### 3.1. The Essential Roles of Matrix Secretion, Bone Mineralization, and Regulatory Hormones in Bone Formation

Newly differentiated osteoblasts play a crucial role in bone formation by synthesizing and secreting the extracellular matrix ECM), which serves as a scaffold for subsequent mineralization. This meticulously crafted ECM undergoes mineralization, transforming into resilient, mineralized bone tissue. The intricate process of ECM secretion and mineralization is orchestrated by various hormones, with PTH playing a central role in maintaining calcium balance and facilitating bone matrix hardening [54]. In addition to hormonal control, several trace elements, such as calcium, magnesium, iron, and phosphorous, are involved in bone metabolism, ensuring the proper remodeling of bones [45].

In zebrafish, collagen and osteocalcin are the proteins important for bone matrix, and their quantities are specifically regulated by BMP signaling, ensuring the optimal environment for bone tissue development [55]. In axolotls, limb regeneration and new bone formation are mediated by PTHrP, which targets osteoblasts and exhibits anabolic effects similar to PTH in humans [56]. This delicate balance underscores the complex interplay between different biological mechanisms and their critical contributions to the robust process of bone formation.

### 3.2. Vascularization and Nutrient Supply

The survival and development of newly formed bone tissues rely on nutrient supply and oxygen through newly formed blood vessels. Hence, VEGF plays a central role during angiogenesis in bone repair [57,58,59]. In zebrafish, VEGF is upregulated in the blastema and surrounding tissues, stimulating the formation of new blood vessels to supply oxygen and nutrients for the development of bone tissues [18].

### 3.3. Remodeling and Strengthening

Followed by the formation and mineralization of the bone matrix, the final process in bone healing involves tissue restructuring into the original bone structure, and this process is regulated by hormones like growth hormones and IGF-1.

The remodeling process ensures the proper function and structural integrity of newly formed bone tissue in harmony with the skeletal system. In axolotls and zebrafish, this process restores full functionality, even after injury without scarring [12,15,60,61,62].

## 4. Hormonal Regulators of Bone Regeneration

### 4.1. Parathyroid Hormone

It is well known that PTH and PTHrP play essential roles in the development of bone and cartilage. Both signal through G-protein-coupled receptors, as PTH regulates the serum calcium and phosphate balance in regulating bone metabolism [63] while PTHrP has a role in development [64] with noteworthy implications for tissue repair and regeneration.

Moreover, PTH is a potent stimulant of bone formation; when administered exogenously, it can also influence bone resorption, depending on its dosage and the frequency of administration. These dual effects are important when considering the potential applications of PTH in bone repair, particularly for treating osteoporosis and managing fractures [65,66,67].

#### 4.1.1. Molecular Mechanism Underlining Bone Remodeling

PTH plays a decisive role in orchestrating both bone resorption and formation, primarily through its interaction with the parathyroid hormone receptor I (PTH1R) located on osteoblastic cells, including osteocytes [68,69]. This interaction triggers various signaling cascades, such as the cyclic adenosine monophosphate/protein kinase A (cAMP/PKA) pathway and the *Wnt*/β-catenin pathway, both of which are essential for bone formation [68,69,70,71]. When PTH binds to PTH1R, it activates the enzyme protein kinase A (PKA) that, in a cascade, phosphorylates and inactivates glycogen synthase kinase-3 beta (GSK-3β) [72]. This suppression leads to β-catenin being retained within the cytoplasm, followed by its nuclear translocation and the activation of *Wnt* genes.

The regulation of *Wnt*/β-catenin signaling by PTH is important not only for the inflammatory phase but is also required during the repair phase of bone tissue physiology. At this stage, the β-catenin activates other transcription factors, including Runx2, which is involved in osteocalcin synthesis and other proteins in the developing bone matrix. However, in experimental models, it has been demonstrated that the phase of PTH administration is crucial because constant exposure to PTH brings anabolic effects to the bone. Therefore, repeated exposure enhances bone formation without overstimulating resorption [73].

It is suggested that PTH administration at regular intervals promotes the proliferation and survival of osteoblasts, thereby encouraging bone formation. This mechanism presents a promising therapeutic approach for treating osteoporosis, aligning with the regenerative processes observed in the axolotl model [74].

This connection describes the potential for leveraging insights from axolotl regeneration to enhance therapeutic strategies for human bone repair. However, recombinant PTH is already employed in human medicine for bone remodeling, demonstrating its utility in regenerative applications. For instance, studies have shown that PTH combined with biomaterials considerably improves bone healing in critical-sized defects. This finding illustrates the promising potential of PTH in orthopedic surgery, where it can be integrated into treatment protocols to enhance bone regeneration and repair [75].

#### 4.1.2. Regulatory Influence on Osteoblast and Osteoclast Activity

PTH plays a crucial role in bone remodeling by stimulating osteoblasts to secrete RANKL, which enhances the formation and activity of osteoclasts [76]. Moreover, parathyroid hormone is an evolutionarily conserved molecule, expressed in specific tissues, playing a major role in processes such as bone repair and skeletal remodeling. Its conservation across various species underscores its fundamental biological significance while revealing the diverse evolutionary adaptations that allow different organisms to leverage PTH uniquely. A critical appraisal of these roles of PTH not only deepens our comprehension of its physiological functions but also opens up potential therapeutic avenues for treating bone-related diseases. However, bone remodeling can never be accomplished without calcium, further necessitating the role of PTH [77]. The transition from an aquatic to a terrestrial lifestyle compelled the acquisition of calcium from external sources through dietary intake. Once absorbed into the bloodstream, calcium must be effectively deposited into the bone to enhance bone mass. This process is critically regulated by vitamin D, which facilitates calcium absorption in the intestines and directs its incorporation into the skeletal system, ensuring proper bone mineralization and maintenance [78].

In mammals, PTH is directly linked with hypocalcemia via the activation of osteoclasts through the RANK/RANKL/OPG (Osteoprotegerin-OPG) pathway. However, its action is irregular, inexplicably stimulating the production of more osteoblasts and bone formation [79]. Moreover, the cyclic administration of PTH can shift the balance towards bone formation, supporting the use of PTH in conditions like osteoporosis [80]. This volatility in mechanistic function could be explained by evolution and highlights the biomedical uses, where these mechanisms can be comprehended and integrated for the enhanced management of bone degeneration and injury.

#### 4.1.3. Translational Application of Human Regenerative Medicine

Many studies have explored the applications of PTH in human bone regeneration; however, its effectiveness remains limited for large bone defects and optimal bone-implant integration [81,82,83]. Employing PTH locally at the wound site through controlled-release polymers, such as biodegradable scaffolds, has been shown to improve bone healing, suggesting promising clinical applications. These advancements, built upon regenerative models like the axolotl, demonstrate that incorporating PTH into local therapies could transform the clinical management of bone repair and regeneration [84].

Clinically, PTH has been effectively utilized to address bone loss conditions, such as osteoporosis, primarily due to its anabolic effects on bone mineral density [85]. The systemic administration of PTH not only stimulates bone formation but also demonstrates efficacy in scenarios that do not respond to conventional treatments, including fracture healing. Extensive studies have revealed that PTH can increase bone mass in critical-sized defects in both animal models and human subjects. These findings highlight its considerable clinical potential for treating non-unions and large bone defects [86]. Its ability to enhance bone regeneration and facilitate healing in challenging cases presents an important avenue for improving patient outcomes in bone repair and regeneration. As research continues to explore the mechanisms and applications of PTH, its role in orthopedic therapies may expand, offering new hope for patients with complex bone-related conditions.

### 4.2. The Role of Calcitonin and FGF23 in Bone Homeostasis, Mineralization, and Regeneration Signaling

Calcitonin and FGF23 are required for the maintenance of bone mass and the mineralization process. They function in conjunction with the *Wnt*/β-catenin and BMP signaling pathways, which play key roles in bone tissue repair [87,88,89].

#### 4.2.1. Calcitonin: A Hypocalcemia Agent with Multifaceted Roles

Calcitonin, primarily secreted by the thyroid gland, is well-known for its hypocalcemic activity, which is achieved by downregulating the activity of osteoclasts and reducing bone resorption, thereby promoting mineralization. This function is critical for maintaining calcium homeostasis and is particularly beneficial during increased bone formation or repair periods [90]. Importantly, calcitonin is not solely an inhibitor; it also acts as a stimulator of osteoblasts by stabilizing β-catenin within the *Wnt*/β-catenin signaling pathway. By suppressing GSK-3β, calcitonin prevents the degradation of β-catenin, thereby activating osteogenic genes essential for bone formation [91].

#### FGF23: Dual Roles in Phosphate Metabolism and Bone Mineralization

FGF23 plays a crucial role in regulating phosphate homeostasis by inhibiting renal phosphate reabsorption and suppressing calcitriol synthesis, thereby influencing bone mineralization [90]. FGF23 and calcitonin work together to maintain bone mineral metabolism by regulating both phosphate and calcium, which are essential for normal bone mineralization.

However, the interactions between these hormones are complex and nuanced. Disruptions in their balance can lead to various bone disorders, such as osteoporosis and osteomalacia, highlighting their significance in human health. For instance, hypophosphatemia and elevated levels of FGF23, commonly observed in hypophosphatemic disorders, impair bone mineralization and can result in rickets [92].

#### 4.2.2. Interactions with Signaling Pathways During Regeneration

##### Calcitonin and *Wnt*/β-Catenin Signaling: A Critical Nexus

The activation of this pathway is complex and does not follow a linear sequence. Other factors, including hormones such as PTH, mechanical stimulation, and non-calcitonin components of the *Wnt*/β-catenin pathway, also play pivotal roles in bone formation, with calcitonin serving a supportive function [91].

However, in zebrafish fin regeneration, it can be seen how calcitonin modulates *Wnt* signaling. During the regeneration process, calcitonin regulates the activity of β-catenin to control cell proliferation to avoid over-proliferation, which may lead to malformations or tumors. This endocrine regulation is important during the proliferative phase of blastema formation, where calcitonin is placed in the position opposite to *Wnt* signaling for proliferation [93]. This highlights the demand for a dynamic balance between calcitonin and *Wnt* signaling during blastema formation. The interplay of hormonal, mechanical, and molecular pathways orchestrates regeneration and bone remodeling, ensuring controlled proliferation, structural integrity, and effective tissue restoration.

##### FGF23, Wnt/β-Catenin, and BMP Signaling: A Complex Relationship

Understanding the role of FGF23 in bone regeneration is complicated by its regulatory influence on the *Wnt*/β-catenin and BMP signaling pathways. Under physiological conditions, FGF23 can activate *Wnt*/β-catenin signaling crucial for osteoblast differentiation and functionality. However, in cases of pathological FGF23 activation, often associated with certain metabolic disorders, this signaling becomes detrimental, leading to disrupted bone mineralization [94].

Both FGF23 and BMP play significant roles in bone formation, with FGF23 having the ability to either enhance or inhibit BMP-induced osteogenesis [92,95,96]. For example, BMP signaling promotes osteoblast proliferation, while markers such as Runx2 and FGF23 can interfere with this pathway. Although the precise mechanisms remain unclear, any disruption in FGF23 biosynthesis or activity can profoundly alter BMP signaling and the outcomes of osteogenesis [97].

This complex relationship highlights that, while FGF23 is essential for maintaining bone homeostasis, its overproduction and dysregulation can negatively impact bone healing. Hence, both calcitonin and FGF23 exhibit multifaceted roles in bone regeneration by modulating the *Wnt*/β-catenin and BMP signaling pathways. These interactions are highly condition-specific and can be influenced by various pathophysiological states. Furthermore, mechanical loading and metabolic cues additionally integrate into these pathways, influencing *Wnt*/β-catenin and BMP signaling to maintain bone integrity and facilitate repair. The interplay between hormonal, molecular, and biomechanical factors emphasizes the complexity of bone remodeling, reinforcing the need for a systems-based approach to study bone regeneration.

Table 2 summarizes the relative functions of calcitonin and FGF23 in bone metabolism, mineralization, and renewal signaling across different organisms. Calcitonin primarily functions as a hypocalcemic hormone, inhibiting osteoclast activity to regulate calcium levels and prevent excessive bone resorption. On the other hand, FGF23 plays an important role in phosphate homeostasis, influencing mineralization and indirectly affecting bone regeneration. Although calcitonin is not directly involved in regeneration, its role in calcium homeostasis may indirectly affect regenerative processes. In contrast, FGF23 contributes to mineral metabolism and has been implicated in tissue regeneration, particularly in zebrafish and mice. Key genes, such as the calcitonin receptor-Like Receptor (CRLR/CALCR), calcitonin receptor (CTR), and FGF23, regulate these processes.

##### Integrating Calcitonin and FGF23 in Regenerative Therapies

Both calcitonin and FGF23 play vital roles in regulating the signaling pathways that control bone remodeling and mineralization, and their interactions with *Wnt*/β-catenin and BMP pathways offer valuable insights for regenerative medicine. The dual functions of calcitonin—acting as both an inhibitor of osteoclasts and a stimulator of osteoblasts—combined with the complex regulatory influence of FGF23 on phosphate metabolism and osteogenesis, highlight the potential of these hormones in therapeutic approaches aimed at enhancing bone regeneration.

Future therapies that target the calcitonin and FGF23 signaling axes could be designed to restore the balance of bone resorption and formation, accelerating recovery from fractures or other bone defects. In particular, therapies that modulate these pathways could be useful for conditions such as osteoporosis, osteomalacia, and other metabolic bone diseases, where bone turnover is disrupted. By fine-tuning these signaling pathways, we could enhance the body’s natural regenerative capacity and promote more efficient healing.

### 4.3. Insulin-like Growth Factors (IGFs) and Growth Hormone (GH)

Insulin-like growth factors and GH are fundamental for bone formation and cell regeneration [103,104]. However, their functions are manifold and often contradictory. Hence, the impact should be discussed in more detail. This section briefly discusses the roles of IGFs and GH in bone growth, repair, and cellular regeneration, highlighting both their positive effects and the associated risks.

#### 4.3.1. IGFs and GH in Bone Growth and Repair: A Complex Interplay

IGFs play a critical role in bone formation by promoting osteoblast proliferation, differentiation, and maturation. Specifically, IGF-I is essential for bone formation and remodeling, binding to its receptor (IGF-I receptor) to activate signaling pathways such as phosphoinositide 3-kinase/protein kinase B (PI3K/Akt) and mitogen-activated protein kinase (MAPK). These pathways regulate bone matrix synthesis and mineralization, contributing to skeletal development and maintenance [105].

Growth hormones also influence the hepatic and local (within bone tissue) production of IGF-I to increase its bone-forming effect [106]. Notably, both IGF-I and GH are beneficial in the physiological process in the organism, but their overproduction or uncontrolled secretion causes certain adverse effects. As elevated levels have been linked to conditions such as acromegaly and certain cancers, where their pro-angiogenic and anti-apoptotic properties contribute to disease progression [107]. Additionally, alterations in IGF-I signaling lead to bone disorders and poor bone quality, highlighting the need for careful applications [108].

#### 4.3.2. Regulation of Cell Proliferation and Differentiation During Regeneration

In tissue regeneration, IGFs play a unique role in regulating the number of cells and various cell types, such as mesenchymal stromal cells/stem cells (MSCs). IGF-I supports MSC self-duplication and guides them to differentiate into osteogenic and chondrogenic lineages needed for tissue remodeling [109]. At the same time, GH promotes these processes by increasing the concentrations of IGF-I and immediately affecting progenitor cells [110].

The effects of IGFs are twofold on cell proliferation as they not only promote it but also accelerate its rate. Although it is necessary for regeneration, excessive proliferative activity can lead to oncogenic responses, especially when impairing the regulatory mechanism [111]. Moreover, dysregulated IGF signaling pathways can lead to fibrosis and tissue dysfunction. Therefore, it must be activated appropriately during tissue regeneration therapies [112].

However, the differentiation decisions elicited by IGFs are relative to the surrounding microenvironments and, therefore, subject to other cues. For example, in adipose tissue, IGF-I can act as an undesired factor in initiating adipogenesis under some circumstances, which will hinder the regenerative processes of other tissues like muscle or bone [113]. Thus, such subtle differentiation attests to the complexity of the process of regeneration that occurs in response to IGF and other growth factors, as well as the need for strict control of the signaling milieu.

#### 4.3.3. Interactions and Challenges Within IGF and GH Signaling Networks

The signaling cascades triggered by IGFs and GH are complex networks involving several intermediaries and feedback regulations. For instance, the PI3K/Akt pathway regulates cell survival and proliferation, but mutations or dysregulation in this pathway can contribute to metabolic disorders, such as insulin resistance [114]. Likewise, the MAPK pathway plays a role in differentiation and growth; however, uncontrolled activation will result in oncogenesis [115].

Interactions between these pathways and other signaling molecules add to the crosstalk, making it complex to decipher. The modulation of IGF and GH signaling is possible, involving interaction with TGF-β and BMPs, exerting synergistic or inhibitory effects on bone and tissue regeneration [116]. Disturbances in these interactions can lead to developmental abnormalities and impaired healing responses due to genetic mutation or environmental stress [113].

The idea of the therapeutic manipulation of these pathways is not without its problems. Although the systemic use of IGFs or GH can be useful in curing growth disorders and promoting repair work, its side effects include fluid retention, arthritis pain, and presumed carcinogenicity [114]. Therefore, efforts continue to be focused on designing chemotherapeutic agents that enhance the beneficial effects of IGF and GH signaling while avoiding potential adverse outcomes.

While IGFs and GH are essential for bone growth, repair, and cellular regeneration, the process is fraught with challenges and potential risks. It is crucial to unveil the modus operandi of their signaling axes to optimize the regenerative approach to tissues without damaging effects [115,117,118]. Nevertheless, more attention should be paid to understanding the interactions of these processes and discovering new ways of targeting IGF and GH with high specificity and efficacy.

## 5. Conclusions

Bone remodeling in humans shares remarkable parallels with the regeneration processes observed in model organisms, such as axolotls and zebrafish. Both systems rely on tightly regulated molecular pathways involving a dynamic interplay of signaling molecules, cellular phenotypes, and environmental cues to achieve structural integrity and functional recovery. While bone remodeling in humans primarily facilitates maintenance and repair, regeneration in axolotls and zebrafish enables the restoration of entire skeletal structures. Understanding these processes at a molecular and cellular level provides valuable insights into the conserved mechanisms of tissue regeneration. These findings offer immense potential for advancing regenerative medicine, particularly in developing novel therapies to enhance bone repair and regeneration in humans.

Advancements in understanding the hormonal regulation of bone regeneration have paved the way for potential therapeutic applications. However, clinical translation remains challenging due to risks such as abnormal tissue development and metabolic disturbances, highlighting the need for further research to refine these approaches. Comparative studies of regenerative models like axolotls and zebrafish reveal evolutionary similarities and provide insights into analogous processes in humans that could be harnessed for regenerative medicine. Yet, the complexity of hormonal and genetic interactions demands caution, as manipulation of these pathways entails inherent risks. Balancing innovation with safety is essential for successful clinical applications.

This review explored the cooperative and antagonistic interactions of hormones and signaling pathways essential to bone repair. This complicated signaling network is responsible for the choreographing of the bone healing process right from the inflammatory phase through the repair and remodeling phases. Understanding these cooperative and antagonistic interactions of hormones and signaling pathways is essential to the bone repair process. The hormones and the subsequent regulation of signaling pathways, predominantly *Wnt*/β-catenin, BMP, and TGF-β, describe the modality of effective tissue remodeling without cranky cellular growth or fibrosis.

Each hormone targets specific genes and proteins during different phases of the regeneration process, ensuring efficiency and proper order. These interactions are valuable for keeping the balance between the formation and differentiation of tissues, ultimately resulting in functional tissue repair without forming scars.

The interplay of hormones and signaling pathways is critical for effective tissue repair, with *Wnt*/β-catenin, BMP, and TGF-β playing pivotal roles in bone regeneration. In addition, while PTH promotes bone formation and has therapeutic value, its bone-resorbing properties must be carefully managed to avoid exacerbating osteoporosis or other bone-related diseases. On the other hand, IGFs are vital for osteoblastogenesis, but their potential to stimulate oncogenic pathways limits their direct application in regenerative medicine.

In addition, the complex roles of calcitonin and FGF23 in bone remodeling, along with their interactions with key signaling pathways, indicate their potential as promising therapeutic targets in regenerative medicine. A deeper understanding of the precise molecular mechanisms governing their influence on bone repair is crucial for developing targeted interventions that enhance bone regeneration and improve clinical outcomes in bone-related disorders. 

### Future Research Directions and Clinical Applications

While there is significant potential for regenerative medicine informed by endocrinological insights, there remain substantial gaps in our understanding that must be addressed in future research to fully explore the roles of lesser-known hormones in bone regeneration. Specifically, the effects of these hormones on critical signaling pathways, such as *Wnt*/β-catenin, BMP, and TGF-β, require further investigation. Additionally, advancing our understanding of hormones like FGF23 and their roles in mammalian systems could open up new therapeutic opportunities.

To facilitate clinical applications, a greater focus on precision medicine is essential to minimize risks such as tumorigenesis and metabolic dysregulation. This may involve developing targeted hormone delivery systems or biomaterials for localized hormonal regulation. Using model organisms like axolotls, which exhibit remarkable regenerative abilities, presents a promising strategy for identifying novel therapies with potential human applications. The primary challenge lies in understanding the complex interactions between hormones and regeneration and developing safe, effective interventions for bone injuries or degenerative diseases with minimal side effects.

## Figures and Tables

**Table 2:** Comparative analysis of the roles of calcitonin and FGF23 in bone homeostasis, mineralization, and regeneration signaling across different organisms.

Organism	Protein	Role in Bone Homeostasis	Role in Mineralization	Role in Regeneration Signaling	Key Genes Involved	Research Insights
Humans	Calcitonin	Regulates calcium levels by inhibiting bone resorption	Helps to prevent excessive bone demineralization	Not specifically involved in regeneration	FGF23	Calcitonin acts as a hypocalcaemic factor, reducing calcium levels by inhibiting osteoclast activity [98]
Humans	FGF23	Regulates phosphate homeostasis by reducing renal phosphate reabsorption	Influences bone mineralization through phosphate regulation	Plays a role in mineral homeostasis, affecting regeneration	CTR, CRLR	FGF23 acts as a phosphaturic hormone, regulating phosphate and vitamin D levels, which are crucial for bone health [99,100]
Zebrafish	Calcitonin	Acts as a hypo calcaemic factor, reducing calcium levels in the body	Downregulates epithelial calcium channels (ECaC) to manage calcium absorption	Plays a role in calcium homeostasis, which could impact bone regeneration	FGF23,	Calcitonin and its receptors are involved in regulating calcium levels in response to environmental calcium concentrations [98]
Zebrafish	FGF23	Regulates phosphate and calcium homeostasis	Involved in bone mineralization by regulating phosphate metabolism	Critical in tissue, including fin and muscle regeneration	FGF23, PTH1R	FGF23 plays a significant role in phosphate and calcium regulation, with expression beginning early in development and continuing into adulthood [101]
Mice	FGF23	Essential for systemic phosphate and calcium balance	Key in mineralization by controlling phosphate levels	Affects bone regeneration through its role in mineral metabolism	FGF23,	FGF23 regulates phosphate and calcium levels, with disruptions leading to marked mineralization defects [102]

## Data Availability

Data are contained within the article.
